# Proportioning optimization of transparent rock-like specimens with different fracture structures

**DOI:** 10.1038/s41598-024-59886-8

**Published:** 2024-04-24

**Authors:** Jie Cui, Junshan Hao, Ping Li, Chao Li, Youliang Zhang, Kuilong Wang

**Affiliations:** College of Civil Engineering and Architecture, Hainan University, Haikou, 570228 China

**Keywords:** Transparent rock-like materials, Failure modes, Brittleness degree, Fracture structures, Proportion optimization, Chemistry, Engineering, Materials science

## Abstract

Clarifying the principles of proportioning optimization for brittle transparent rock-like specimens with differential fracture structures is crucial for the visualization study of the internal fracture and seepage evolution mechanisms in rock masses. This study, utilizing orthogonal experimental methods, uncovers the influence mechanisms, extents, and patterns by which the ratios of resin, hardener, and accelerator, along with the freezing duration, impact the mechanical characteristics of transparent rock-like specimens. Notably, it was observed that as the accelerator ratio and freezing time are increased, there’s a general decline in the uniaxial compressive strength, tensile strength, and elastic modulus of the specimens. In contrast, an increase in the hardener ratio initially leads to an enhancement in these mechanical properties, followed by a subsequent decrease. Under uniaxial compressive loading, the specimens exhibit four typical modes of failure: bursting failure, splitting failure, single inclined plane failure, and bulging failure. As the hardener and accelerator ratios increase, the mode of failure gradually shifts from bulging to bursting, with freezing time having a minor overall impact on the evolution of failure modes. The study proposes a method for inducing random three-dimensional closed fractures within the specimens and further clarifies the principles for optimizing the proportions of specimens with different fracture structures, such as intact, embedded regular, and random three-dimensional fractures. This research facilitates the in-depth application of transparent rock-like materials in various scenarios and provides theoretical guidance and technical support for visualizing the evolution of fracture and seepage characteristics within the fractured rock mass.

## Introduction

Rock-like material experiments are an early-developed and widely-used research method for studying the mechanical properties of rock masses. In rock mechanics experiments, the selection and optimization of proportions for rock-like materials have always been an important part of model testing research. Rock-like materials have the advantages of low raw material costs, simplicity in production, and easily adjustable experimental conditions and schemes. They also reflect to a certain extent the objective failure characteristics of rocks, which is why rock-like materials have been extensively researched in indoor experimental studies of rock engineering.

Traditional rock-like materials primarily use cement and gypsum as binders, with quartz sand and barite powder as aggregates, and are widely applied in small-scale fracture rock block tests and large-scale cavern model experiments^1–4^. Specimens prepared with traditional rock-like materials are similar to real rocks in terms of materials and structure, especially clastic rocks like sandstone. This similarity forms the basis for studies on the progressive failure of rock masses and their hydromechanical coupling characteristics^5–8^, effectively addressing several issues such as the difficulties in sampling and processing real rock masses, size limitations of high-strength rock samples due to equipment load capacity, and the inability to accurately control single-variable factors like rock structure. Therefore, considering the degree of structural and mechanical characteristic similarity, traditional rock-like materials are the optimal choice for preparing rock-like specimens. However, the presence of many three-dimensional fractures in rock masses, enveloped in the rock medium space, and their controlling mechanisms on the progressive failure of rock masses under load, as well as the seepage characteristics of fracture water in water-rich strata and its promoting effect on fracture propagation, are constrained by the non-visual nature of traditional rock-like materials, making it impossible to monitor and capture intuitive images. Given the current limitations of indirect monitoring methods, such as the precision of acoustic emission in locating fracture expansion, and the real-time nature, applicable sample size, shape, and loading conditions of CT scan technology, the study of transparent rock-like materials becomes particularly important in revealing the internal fracture expansion and seepage evolution processes within the specimens.

Transparent rock-like materials are currently mainly used in research on the mechanisms of three-dimensional fracture expansion in rock masses, hydraulic fracturing, shear seepage characteristics of rough joint surfaces, and rock blasting characteristics. They utilize their transparency to intuitively reveal the internal fracture expansion and seepage evolution patterns in rocks. Therefore, to ensure the similarity in mechanical behavior and transparency between transparent rock-like materials and real rocks, many scholars are dedicated to enhancing their brittleness and transparency, thereby advancing related research. Dyskin^9^ was one of the first to use transparent materials classified as toughened materials. In an environment of −17 ℃, the material's tensile and compressive strength ratio was only 1/3. Using this material, the mechanism of three-dimensional crack expansion under uniaxial compression was studied. Sahouryeh et al.^10^ used the same material to conduct experimental and analytical studies on the expansion of three-dimensional cracks under biaxial compression. Huang M. and Huang K.^11^, as well as Wong et al.^12^, used polymethyl methacrylate (PMMA), which has a tensile-to-compressive strength ratio of only 1/3, far from meeting the brittleness characteristics of rock-like materials. They studied the expansion, evolution, and coalescence mechanisms of three-dimensional surface cracks with different depths and angles under uniaxial compression conditions. Guo et al.^13^ developed a type of unsaturated resin material that exhibits good transparency and brittle fracture characteristics at low temperatures, particularly at −30 ℃ where its tensile-to-compressive strength ratio can reach 1/5. This material's brittleness is superior to polymethyl methacrylate. They revealed the expansion and coalescence process of parallel three-dimensional fracture groups under uniaxial compression conditions. Shi et al.^14^ used an unsaturated polyester resin to prepare the specimens and by continuously adjusting the ratio of additives, the specimen tensile-to-compressive strength ratio could be as high as 1/5 and even 1/7 at −50 °C. Their research revealed the expansion and coalescence process of closed three-dimensional cracks under uniaxial compression conditions, as well as the influence of crack inclination angle, area, friction coefficient, and spacing of composite cracks on the mechanical properties, crack expansion, and coalescence process. Sun et al.^15^, by altering the proportions of the hardener and accelerator, and simultaneously controlling curing temperatures and other maintenance conditions, developed a new type of unsaturated resin material. This material can achieve a tensile-to-compressive strength ratio of 1/5 at room temperature. After being stored at −60 ℃ for 48 h, its tensile-to-compressive strength ratio can reach 1/7, or even 1/8. Based on this material, Zhu, Lin and Sun et al. revealed the effects of fissure length, inclination, spacing, and rock bridge angle on the crack expansion and coalescence modes of single or multiple three-dimensional fissure and the strength of the specimens under uniaxial compression^16–18^. Zhu et al.^19^ used this material to conduct hydraulic fracturing experiments on single fracture specimens under uniaxial and biaxial compression conditions, and revealed the effects of different osmotic pressures and crack forms on the initiation, coalescence, and peak strength of elliptical opening-type cracks. Fu et al.^20^ developed a rock-like material formed by the reaction of a new type of unsaturated polyacrylate resin and hardener, which exhibits good brittle fracture characteristics at lower temperatures. This material has good transparency, with a tensile-to-compressive strength ratio of 1/6.6. Based on this material, the rupture evolution laws and mechanisms of fracture specimens under uniaxial and biaxial compression conditions in the absence and presence of hydraulic pressure were investigated^21–25^. Ge and Xu^26^ developed a transparent hard rock-like material, which is made from a mixture of saturated rosin solution, epoxy resin, and hardener. The tensile-to-compressive strength ratio of this material can reach as high as 1/13.2, and its physical and mechanical properties are similar to those of hard rocks. This material has been applied in blasting model experiments. Huang et al.^27^ used 3DP (3D printing) resin to create transparent specimens with natural joint surface morphologies. They enhanced the mechanical properties of the 3DP resin through a freezing method to more accurately replicate the characteristics of hard rocks. Hu et al.^28^ selected E-44 type epoxy resin as the aggregate, triethanolamine as the hardener, and added alcohol rosin saturated solution (RSS) to adjust brittleness. The transparency, rock-like properties and strength tunability of transparent rock bodies with different ratios were investigated.

Furthermore, with the emergence of problems like water inrush and instability in rock bodies in water-rich geological environments, and the improvement of experimental conditions, many scholars have embarked on more in-depth explorations of the failure mechanisms of fractured rock bodies under the coupled effects of high water pressure and engineering excavation disturbances. Based on transparent rock-like materials, Zhu et al.^19^ and Fu et al.^25^ studied the progressive damage mechanisms and patterns of three-dimensional crack tips under hydromechanical coupling. Regarding the shear-flow coupling characteristics of fractures, some researchers^29,30^ used transparent acrylic resin and gypsum to create the upper and lower parts of shear samples with the same surface morphology as natural fractures. They conducted shear-seepage coupling experiments, and, with the help of tracer distribution, revealed the evolution of flow channels between rough fracture walls during shearing and the associated nonlinear fluid flow characteristics.

In summary, the visualization capabilities of transparent rock-like materials are highly favored by scholars, and their brittleness and transparency are continually improved through measures such as low-temperature freezing, optimization of the proportions of hardeners, accelerators, rosin, and other resin additives. However, the optimization of the properties of transparent rock materials still lacks systematic research that provides process demonstrations and conclusive guidance, which undoubtedly hinders further optimization and in-depth application in various scenarios. Moreover, many studies merely base the similarity argument between transparent rock-like materials and real rocks on single or partial indicators such as uniaxial compressive strength, stress–strain curve characteristics, failure modes, or tensile-to-compressive strength ratios. This approach might lead to significant errors in experimental results, as the fracture characteristics of transparent rock-like materials play a crucial role in the evolution of three-dimensional fracture expansion and shear seepage channels. Additionally, research on the mechanical properties of three-dimensional fracture transparent rock-like specimens is limited to the category of regularly filled fractures. There is a lack of appropriate specimen preparation technology for closed random fractures, which are widely developed in hard rocks, thereby restricting the progress of related studies.

This article involves the preparation of transparent rock-like materials using unsaturated resin, hardener, and accelerator, with an enhancement of their mechanical properties through low-temperature freezing. A 3-factor, 5-level orthogonal experiment was conducted, focusing on the hardener ratio, accelerator ratio, and freezing time as influencing factors. Based on the results of uniaxial compression and Brazilian split tests of transparent rock-like specimens, the study reveals the mechanisms, extents, and patterns of how these factors affect the mechanical properties of the specimens. It summarizes the typical failure modes of transparent rock-like specimens under various proportioning conditions and, along with the compressive-tensile strength ratio, systematically evaluates the similarity of these materials in simulating rock mechanical behavior. Finally, based on the experimental results with intact and fractured specimens, the study clarifies the proportioning optimization principles for different fracture structure specimens, including intact, embedded regular three-dimensional filled fractures, and random three-dimensional closed fractures. This research advances the in-depth application of transparent rock-like materials in multiple scenarios and provides theoretical guidance and technical support for the visualization study of the internal fracture and seepage evolution processes in the fractured rock mass.

## Specimen preparation and experimental design

### Selection of raw materials

The raw material selected in this article is transparent unsaturated epoxy resin, which has the advantages of fast curing time and high transparency. Cobalt octoate is chosen as the accelerator and phenolsulfonic acid as the hardener, to adjust and improve the resin's brittleness characteristics. The detailed parameters of the raw materials are shown in Table 1.

**Table 1:** Basic parameters of raw materials.

Name	Physical image	Type	Molecular formula	molecular mass (g/mol)	Density (g/cm^3^)
Resin		Unsaturated epoxy resin	(C_11_H_12_O_3_)_n_	455	1.2
Hardener		Phenolsulfonic acid	C_6_H_6_O_4_S	174.17	0.9
Accelerator		Cobalt octoate	C_14_H_22_CoO_4_	313.25	1.2

### Specimen preparation method

**Figure 1:**
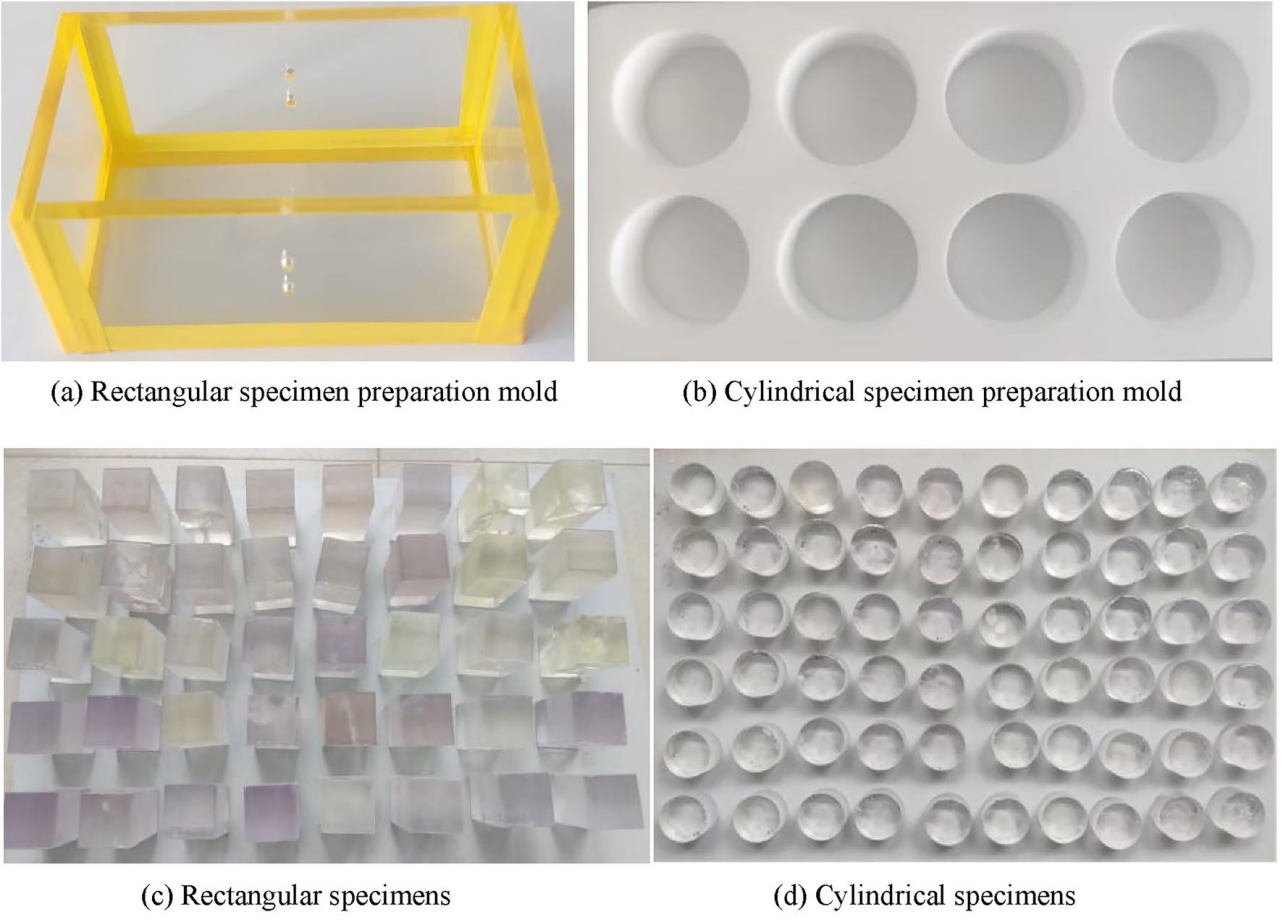
Specimen preparation molds and finished specimens.

Based on the results of trial specimen preparation, the specimen preparation method was continuously refined and optimized to reduce the impact of the preparation method on the transparency and homogeneity of the specimens. The specific specimen preparation steps are as follows:

1. Preparation of raw materials: Prepare the resin, hardener, and accelerator required for the experiment according to the material types listed in Table 1.
2. Mold preparation: Rectangular and cylindrical molds are used to prepare rectangular specimens for the uniaxial compression test and cylindrical specimens for the Brazilian split test, respectively. The rectangular mold is composed of 5 pieces of 5 mm thick transparent acrylic boards, including two rectangular boards each of 50 mm × 60 mm and 50 mm × 100 mm, and one 60 mm × 110 mm rectangular board. These are taped together to form a rectangular mold box with an open top and internal dimensions of 50 mm × 50 mm × 100 mm, as shown in Fig. 1a. The mold for preparing cylindrical specimens is custom-made from polytetrafluoroethylene (PTFE) material, containing several cylindrical grooves of Φ50 mm × 25 mm. This material does not bond with the resin material, making it easy to demold later, as shown in Fig. 1b. For the preparation of rectangular specimens, a transparent mold is used to facilitate the real-time observation of the fracture position and specimen preparation effect during the preparation process of the specimens with three-dimensional fractures.
3. Specimen Preparation: According to the designed raw material proportions, specific weights of resin, hardener, and accelerator are separately measured using a beaker and a small measuring cup. First, the pre-weighed accelerator is poured into a beaker containing resin. It is stirred in a fixed direction for about two minutes. Once the resin becomes transparent and clear, it is left to stand for about a minute, allowing any bubbles generated during stirring to dissipate on their own. Then, the pre-weighed hardener is slowly poured into the beaker, stirring gently along the fixed direction to minimize the generation of excess bubbles. After the curing agent and resin have completely blended, they are poured into the mold using a glass rod for further processing.
4. Specimen Demolding: After the specimen has solidified and returned to room temperature, carefully remove the mold to prevent damage to the edges and corners of the specimen. The demolded square and cylindrical specimens are shown in Fig. 1c,d, respectively. Number the specimens after demolding. There are a total of 25 groups of specimens, with 3 specimens made for each group, numbered as follows: x-1, x-2, x-3 (where x ranges from 1 to 25).
5. Specimen Curing: Use sandpaper to smooth out any uneven areas on the specimens. Then, place the specimens in a −25 ℃ low-temperature refrigerator for curing, and retrieve them before conducting the test.

### Experimental design

The orthogonal experimental method is adopted to study the influence of raw material ratios and freezing time on the mechanical characteristics of specimens. The orthogonal experimental design uses the mass percentage of hardener to resin (hardener ratio A), the mass percentage of accelerator to resin (accelerator ratio B), and the freezing time C as the three factors of the orthogonal experiment, with each factor set at 5 levels. Preliminary exploratory experimental results show: when the A is less than 0.5, the specimens cannot cure; when the A is more than 2.0, the transparency of the specimens significantly decreases. When the B is less than 0.2, the effect of the accelerator is weak; when the B is more than 1.0, the curing process of the specimens is intense, releasing a large amount of heat, and random defect-type damages occur, making the specimen forming process unstable and difficult to form a intact specimen. When the freezing time is less than 6 h, the specimens exhibit obvious toughness; after more than 48 h, there is no significant difference in the mechanical properties of the specimens. Therefore, the ranges of the A, B, and C are set to 0.5 ~ 2.0, 0.2 ~ 1.0, and 6 ~ 48 h, respectively, with the levels of each factor set as shown in Table 2.

**Table 2:** Orthogonal test factor level settings for transparent rock-like materials.

Factor level	A	B	C	Level representation
1	0.67	0.33	6	Low
2	1.0	0.5	12	Lower
3	1.3	0.67	18	Medium
4	1.6	0.83	24	Higher
5	2.0	1.0	48	High

The freezing temperature is set at -25 ℃. The freezing temperature during the experiment is a fixed quantity, and no changes were made to the temperature.

This experiment adopts a 3-factor 5-level orthogonal design scheme L25 (5^3^), designing 25 sets of ratio combination experiments. Each set of experiments contains 3 specimens, and the ratio combinations are shown in Table 3.

**Table 3:** Orthogonal design combination scheme for transparent rock-like materials.

Group No	A	B	C	Group No	A	B	C	Group No	A	B	C	Group No	A	B	C	Group No	A	B	C
1	1	1	3	6	2	1	5	11	3	1	1	16	4	1	1	21	5	1	1
2	1	2	5	7	2	2	2	12	3	2	2	17	4	2	4	22	5	2	2
3	1	3	4	8	2	3	1	13	3	3	3	18	4	3	3	23	5	3	3
4	1	4	2	9	2	4	3	14	3	4	4	19	4	4	2	24	5	4	4
5	1	5	1	10	2	5	4	15	3	5	5	20	4	5	5	25	5	5	5

## Sensitivity analysis of material proportion factors

### Basic mechanical parameters of the specimens

**Figure 2:**
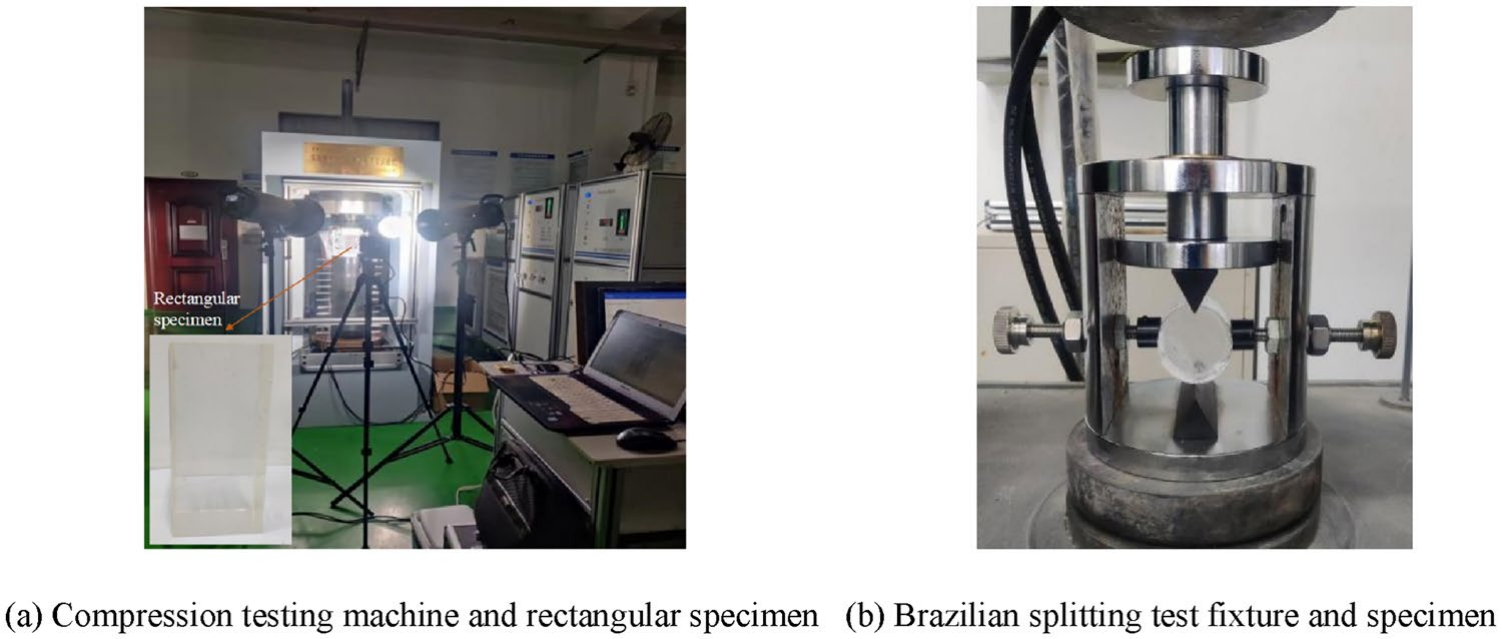
Experimental apparatus and transparent rock-like specimens.

**Figure 3:**
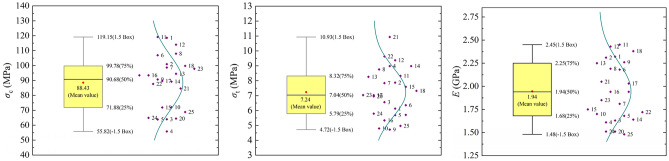
Statistical distribution of mechanical parameters of transparent rock-like specimens.

For the 25 ratio combinations of the orthogonal design, uniaxial compression tests and Brazilian split tests were conducted sequentially on transparent rock-like specimens. The test apparatus and specimens are shown in Fig. 2. The statistical distribution characteristics of the uniaxial compressive strength *σ*_c_, tensile strength *σ*_t_*,* and the tangent modulus of elasticity *E* at 50% of the peak compressive strength are shown in Fig. 3. The test results show that the basic mechanical parameters of transparent rock-like materials under different ratios exhibit significant differences. Specifically, the range of uniaxial compressive strength is 55.82 to 119.15 MPa, the range of tensile strength is 4.72 to 10.93 MPa, and the modulus of elasticity ranges from 1.48 to 2.45 GPa. Therefore, it is evident that varying raw material ratios and freezing times can lead to significant differences in the mechanical characteristics of transparent rock-like materials, especially in terms of uniaxial compressive strength and tensile strength. Thus, further clarifying the degree and pattern of influence of each ratio factor on the mechanical properties of the materials is of great significance for the preparation of transparent rock-like specimens with different brittleness characteristics.

### Sensitivity analysis of factors affecting mechanical parameters

To reveal the degree and pattern of the influence of various factors on the mechanical properties of transparent rock-like specimens, a sensitivity analysis of the basic mechanical parameters of the specimens was conducted. According to the theory of orthogonal experiments, the mechanical parameters at the same level of each factor are averaged, and the range is the difference between the maximum and minimum values of the mechanical parameters at each level. A larger range indicates that the different levels of that factor produce more significant differences, making it an important factor with a significant impact on the experimental results. Let *y*_*i*_ (*i* = 1, 2, …, 5) represent the average mechanical parameter value at level *i* of a factor. For example, in the analysis of the range of *σ*_c_ under the influence of the hardener ratio, *y*_*i*_ represents the average *σ*_c_ when the hardener ratio is at level *i*, under different accelerator ratios and freezing times. The results of the range analysis of the basic mechanical parameters of transparent rock-like specimens are shown in Table 4.

**Table 4:** Range analysis of the mechanical parameters of transparent rock-like specimens.

Mean values of parameters	*σ*_*c*_/MPa			*σ*_*t*_/MPa			*E*/GPa		
	A	B	C	A	B	C	A	B	C
y_1_	80.64	104.54	93.81	7.05	7.41	7.81	1.91	2.19	2.06
y_2_	95.25	98.18	89.92	6.50	8.35	8.31	2.03	2.06	1.90
y_3_	97.74	92.81	100.27	8.50	7.59	6.69	2.10	2.06	2.21
y_4_	88.16	78.81	75.96	6.47	6.84	6.63	1.96	1.79	1.70
y_5_	80.77	68.22	82.61	7.67	6.00	6.75	1.72	1.62	1.84
Range	17.10	36.32	24.31	2.04	2.35	1.67	0.38	0.57	0.51

**Figure 4:**
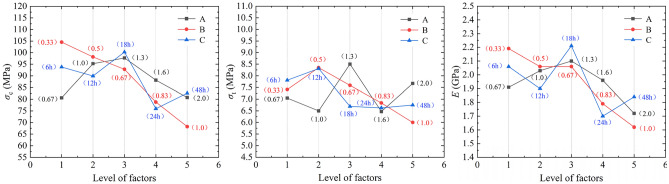
Influence of various factors on the mechanical properties of transparent rock-like specimens.

Table 4 shows that the factors affecting the *σ*_c_ and *E* of transparent rock-like specimens in descending order of impact are accelerator ratio, freezing time, and hardener ratio; for *σ*_t_, the order is accelerator ratio, hardener ratio, and freezing time. It can be observed that the accelerator ratio is the most significant factor affecting the mechanical characteristics of transparent rock-like specimens. To more intuitively display the influence of each factor on the mechanical properties of transparent rock-like specimens, a chart depicting the variation of mechanical parameters at different levels of each factor was created, as shown in Fig. 4. From the chart, it is evident that with the increase in accelerator ratio and freezing time, the *σ*_c_, *σ*_t_, and *E* of the transparent rock-like specimens generally show a decreasing trend. With the increase in hardener ratio, *σ*_c_ and *E* generally show an increasing and then decreasing trend, while the impact of hardener ratio on *σ*_t_ shows fluctuations with a range of 2 MPa, but overall still presents an increasing and then decreasing trend.

To gain a deeper understanding of the influence of various factors on the *σ*_c_, *σ*_t_ and *E* of transparent rock-like specimens, the impact mechanisms of these factors on the mechanical properties of the specimens are further revealed from the perspective of raw material reaction molding and specimen structural characteristics. The higher the content of the hardener, the faster the curing speed, but excessively high levels of the hardener ratio can lead to a rushed and insufficient curing process of the resin. With the increase in the hardener ratio, *σ*_c_ and *E* gradually increase initially. This is because the increase in the hardener ratio enhances the viscosity and epoxy value of the material, thereby strengthening the curing effect and consequently enhancing the *σ*_c_ and *E* of the transparent rock-like specimens, with the impact being quite direct. As the hardener ratio further increases, *σ*_c_ and *E* decrease, which is attributed to the excessive amount of hardener causing incomplete curing reactions in the resin, leading to an increase in internal bubbles in the specimens. The impact of the hardener ratio on *σ*_t_ is characterized by fluctuations; *σ*_t_ reaches its maximum value when the hardener ratio is 1.3, and overall, the impact on *σ*_t_ also shows an increasing and then decreasing trend. Therefore, it is evident that the impact of the hardener ratio on the mechanical properties of the specimens exhibits a clear peak and turning effect, that is, when the curing ratio is 1.3, the specimens exhibit stronger mechanical behavior. In the reaction process of resin, the accelerator can cause changes in active groups, thereby accelerating the cross-linking process and enhancing the curing rate of the resin. With the increase in the accelerator ratio, *σ*_c_, *σ*_t_, and *E* generally show a downward trend. This is because the increase in the amount of accelerator accelerates the curing degree of the specimen. The specimen preparation process shows that as the accelerator ratio increases, a large amount of heat is generated during the curing process, which induces the formation of defect-related damage within the specimen, such as small cracks, leading to the deterioration of the mechanical properties of the specimen. The specimens were subjected to freezing treatment at −25 ℃ to physically reduce their viscosity and increase their brittleness. Compared to the hardener ratio and accelerator ratio, the influence of freezing time on the mechanical properties of the specimens is relatively small. As the freezing time increases, the mechanical parameters of the specimens generally show a decreasing trend. This is mainly because as the freezing time increases, the effect of internal defects in the specimens is magnified. That is, under the action of force, internal micro-cracks and bubbles in the specimens are more likely to induce structural fracturing, leading to an overall decrease in mechanical properties. However, based on the fluctuations in data points, it can be seen that when the freezing time reaches 18 h, the *σ*_c_ and *E* of the specimens are at their maximum values, and when the freezing time reaches 12 h, *σ*_t_ is at its maximum. This indicates that under a combined effect, when the freezing time is between 12 and 18 h, the mechanical properties of the specimens show a certain degree of enhancement. However, as the freezing time continues to increase, the weakening effect of pores and cracks intensifies, and the combined impact leads to a fluctuating deterioration trend in the mechanical properties of the specimens.

## Brittle characteristics and failure modes

**Figure 5:**
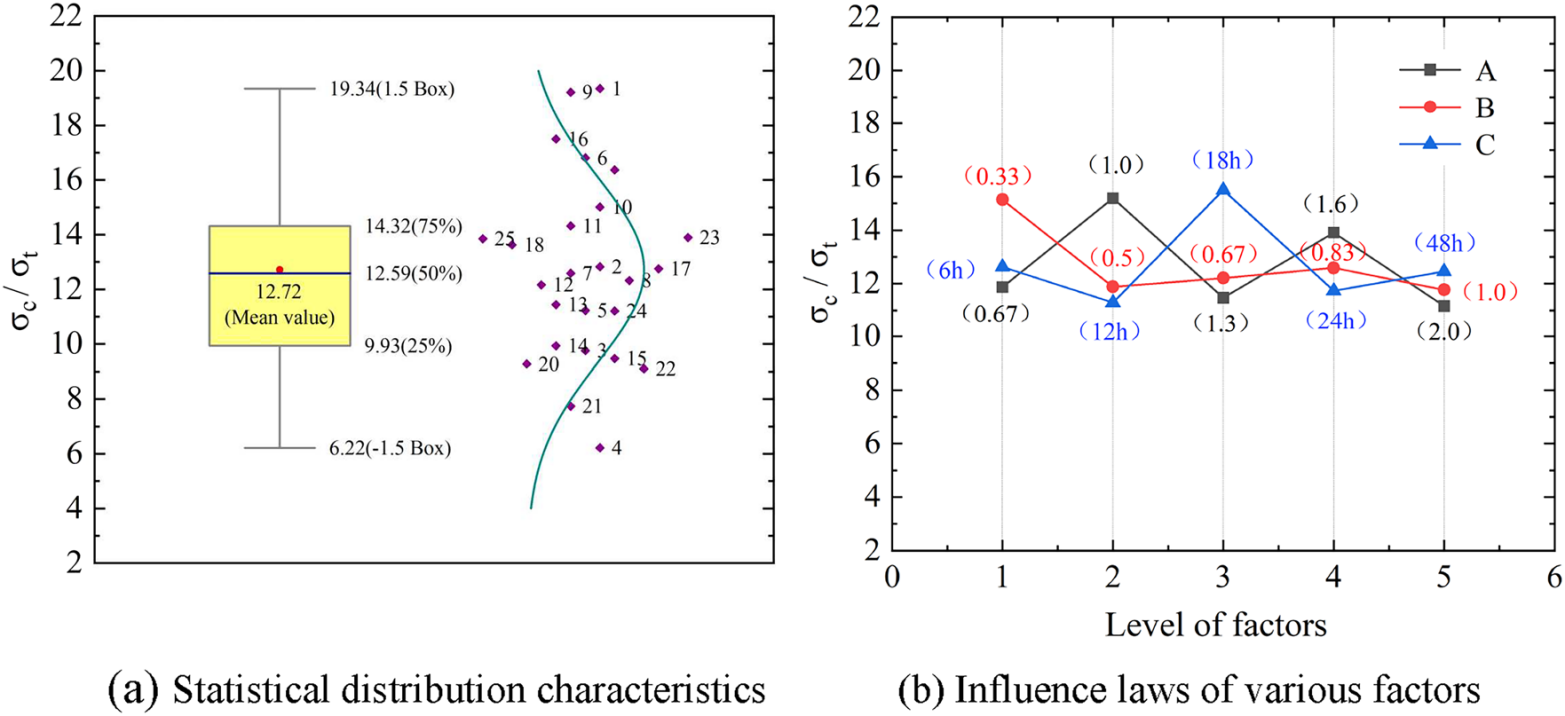
Analysis results of the compressive-tensile strength ratio of transparent rock-like specimens.

Brittleness is an important factor affecting the failure mechanism and characteristics of rocks. When using rock-like materials to study the failure mechanisms and characteristics of rocks, the similarity in brittleness is a key indicator for evaluating the resemblance between rock-like specimens and the original rock. The application fields of resin materials generally favor the toughness, wear resistance, and other properties of resin materials. However, when applied to rock-like materials, their transparency is often utilized to study the expansion characteristics of three-dimensional fractures. Therefore, many scholars are dedicated to researching how to enhance the brittleness index, i.e., the compressive-tensile strength ratio, to ensure its similarity to real rock materials. To compare with previous research results, the compressive-tensile strength ratio is used to quantitatively evaluate the brittleness characteristics of transparent rock-like specimens. The statistical distribution characteristics of the compressive-tensile strength ratio of transparent rock-like specimens are shown in Fig. 5a, with the distribution range of the ratio being 6.22 to 19.34. Compared to the length of the range interval of the compressive-tensile strength ratio, which is 13.12, the range analysis results (Table 5) show that the hardener ratio, accelerator ratio, and freezing time have a comparable impact on the compressive-tensile strength ratio, exhibiting a fluctuating influence pattern, as shown in Fig. 5b. Based solely on the numerical values of the compressive-tensile strength ratio, the prepared transparent rock-like specimens can well meet the range requirements of the compressive-tensile strength ratio for brittle rocks. However, the experimental results show that transparent rock-like specimens with higher compressive-tensile strength ratios tend to exhibit non-brittle failure modes. Therefore, to reasonably evaluate the similarity of transparent rock-like materials in simulating the mechanical behavior of brittle rocks, a comprehensive analysis combining the compressive-tensile strength ratio and the failure mode of the specimens should be conducted.

**Table 5:** Range analysis of the compressive-tensile strength ratio of transparent rock-like specimens.

Factor	*σ*_c/_*σ*_t_					
	y_1_	y_2_	y_3_	y_4_	y_5_	Range
A	11.87	15.19	11.47	13.91	11.16	4.03
B	15.14	11.88	12.21	12.59	11.77	3.38
C	12.62	11.29	15.50	11.73	12.45	4.22

Under uniaxial compression load, transparent rock-like specimens exhibit four typical failure modes, namely bursting failure, splitting failure, single inclined plane failure, and bulging failure. The uniaxial compression stress–strain curves, macroscopic failure modes, and microscopic structural characteristics of the fracture surfaces of the specimens are shown in Table 6.

**Table 6:**
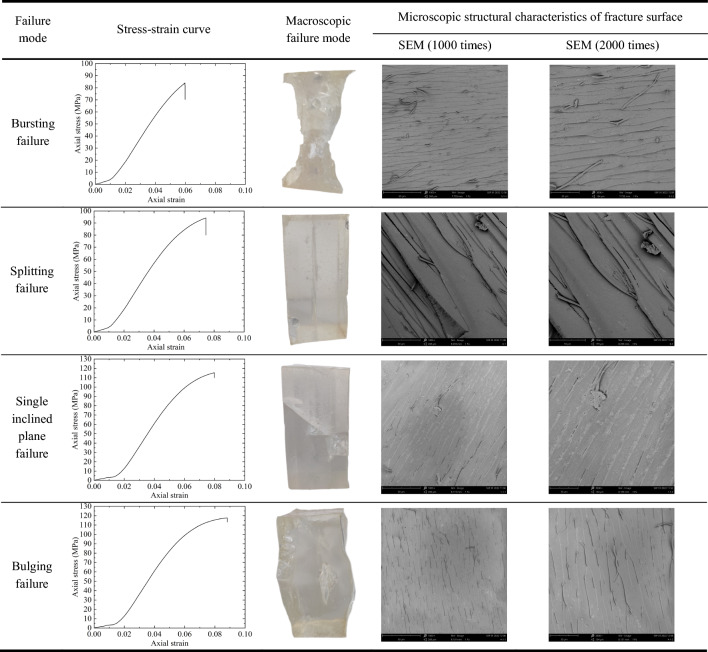
Typical failure modes of transparent rock-like specimens under uniaxial compression condition.

Table 6 shows that under uniaxial compression conditions, the stress–strain curve of transparent rock-like specimens exhibits evolutionary characteristics of an upward concave section, a linear section, a downward concave section, and a steep drop section. These correspond respectively to the initial compaction stage, linear deformation stage, crack development stage, and failure stage of the specimens, which is similar to the failure process of real rocks, and its failure modes can better simulate some failure characteristics of common rocks, as shown in Table 7.

**Table 7:**
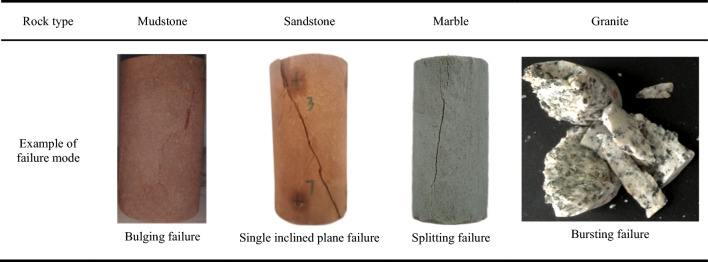
Examples of real rock failure modes under uniaxial compression.

Specimens exhibiting bursting failure, after undergoing a brief initial compaction phase, enter the linear deformation stage. They exhibit instantaneous brittle bursting failure without a noticeable crack development stage. At the moment of failure, fragments of the specimen are violently ejected. The fracture surface features a distinct roughness, with noticeable dimples and tear patterns observed near the crack lines, along with the formation of numerous fine root-like branches. This indicates a significant stress dispersion phenomenon, and the formation of the fracture surface is intense. Specimens exhibiting splitting failure, after a relatively short initial compaction phase, also enter the linear deformation stage. They then progress to the crack development stage, where cracks initiate and gradually propagate throughout the entire specimen due to local micro-cracks or pores, leading to brittle splitting failure. The surfaces of the split fractures tend to be smooth locally, with distinct crack lines developing in a straight line. The phenomenon of stress dispersion disappears, presenting typical brittle fracture striations. The formation of the fracture surface is crisp and straightforward. Specimens exhibiting single inclined plane failure have a noticeably elongated initial compaction phase. After undergoing the linear deformation stage, they gradually form a steeply inclined failure surface. The formation of this failure surface is characterized by a longer crack development stage. Once the fracture surface becomes continuous, the specimen fails, and the entire failure process of the specimen shows a certain degree of plastic deformation. On the fracture surface, the crack lines become dense, the number of stress bands increases, and there are no obvious tears. The stress dispersion effect is apparent, and the formation of the fracture surface is gentle. Specimens exhibiting bulging failure show typical plastic deformation. After a relatively long initial compaction phase, the specimens enter the linear deformation stage and then progress to the plastic deformation stage. As the specimens bulge, tearing occurs in the middle of the specimens, leading to failure. The fracture surfaces of the specimens have dense crack lines in a discontinuous and curved form, with a significant stress dispersion effect. The formation of the fracture surface is gentle.

**Figure 6:**
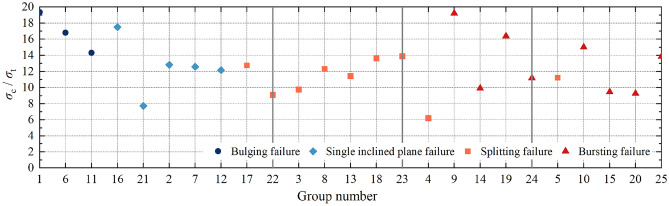
Distribution characteristics of compressive-tensile strength ratio of transparent rock-like specimens with different failure modes.

The compressive-tensile strength ratio of transparent rock-like specimens under different ratios and their corresponding failure modes are shown in Fig. 6. Figure 6 shows:

1. Bulging failure occurs under conditions of low accelerator ratio and medium or lower hardener ratio. Single inclined plane failure occurs under conditions of low accelerator ratio and higher or high hardener ratio, or lower accelerator ratio and medium or below hardener ratio. Splitting failure occurs under conditions of lower accelerator ratio and higher or high hardener ratio, or medium accelerator ratio and any hardener ratio, or higher or high accelerator ratio and low hardener ratio. Bursting failure occurs under conditions of higher or high accelerator ratio and lower or above hardener ratio.
2. There are more specimens exhibiting splitting failure, followed by bursting failure, and then single inclined plane failure and bulging failure. As the levels of hardener ratio and accelerator ratio increase, the failure mode gradually transforms from bulging failure to bursting failure. Overall, the accelerator ratio is the main factor controlling the failure mode of the specimens, with the increase of the accelerator ratio, the failure mode is gradually transformed from plastic to brittle failure; under the same level of accelerator ratio, the hardener ratio accelerates the process of transformation of the failure mode to brittle failure. The influence of freezing time on the overall evolution trend of the failure mode is relatively small.
3. The range of compressive-tensile strength ratios of the transparent rock-like specimens is 6.22 to 19.34, which falls within the range of compressive-tensile strength ratios for real rocks, i.e. 2.7 to 39^31^. Among them, specimens with bulging failure show a significant yield hardening effect and higher compressive strength, with the compressive-tensile strength ratio consistently above 14.0, with an average value of 16.82; The compressive-tensile strength ratios of both single inclined plane failure and splitting failure specimens fluctuated above and below 12.0; The mean value of the compressive-tensile strength ratio of the bursting failured specimens is 13.04, with a relatively large fluctuation range of 9.27 to 19.21.

## Proportioning optimization of different fractured specimen

The greatest advantage of transparent rock-like materials lies in their transparency. Currently, transparent rock-like materials are mainly used in research on the crack propagation mechanisms and modes of rock with three-dimensional fractures under loading or hydraulic pressure, as well as the coupled mechanisms of fracture surface shear and seepage flow. For different research objectives, based on the above findings of this article, the ratio of transparent rock-like materials should be further optimized and clarified. This ensures the rational selection of the ratio for the targeted transparent rock-like specimens, thereby guaranteeing the success rate of specimen preparation.

The selection of the ratio for intact transparent rock-like specimens can be based on the conclusions of the previous studies. By considering the comprehensive characteristics of basic mechanical parameters, failure modes, and the compressive-tensile strength ratio, a transparent rock-like material ratio similar to the mechanical properties of the target rock can be chosen to prepare rock-like specimens, and to carry out the related studies, such as the study of shear seepage and blasting characteristics of the rock mass^26,27,29,30^. By optimising the comprehensive similarity between intact transparent rock-like materials and real rocks, a more accurate representation of shear fracture, seepage and blasting characteristics of  the rock mass can be achieved. Therefore, selecting transparent rock-like materials with mechanical properties similar to the target rock is crucial for the accuracy of the experimental results, rather than merely pursuing their visualization capabilities or similarity in a single mechanical indicator.

## Regular fractured specimens

Three-dimensional fracture networks are extensively developed within rock masses and cannot be directly observed during their expansion and penetration under load conditions. Additionally, fracture networks typically develop in harder rocks, and the control effect of fractures is significantly manifested in hard and brittle rocks. Hence, enhancing the brittleness of transparent rock-like materials is key to accurately approximating the failure mechanisms of the fractured rock mass.

**Figure 7:**
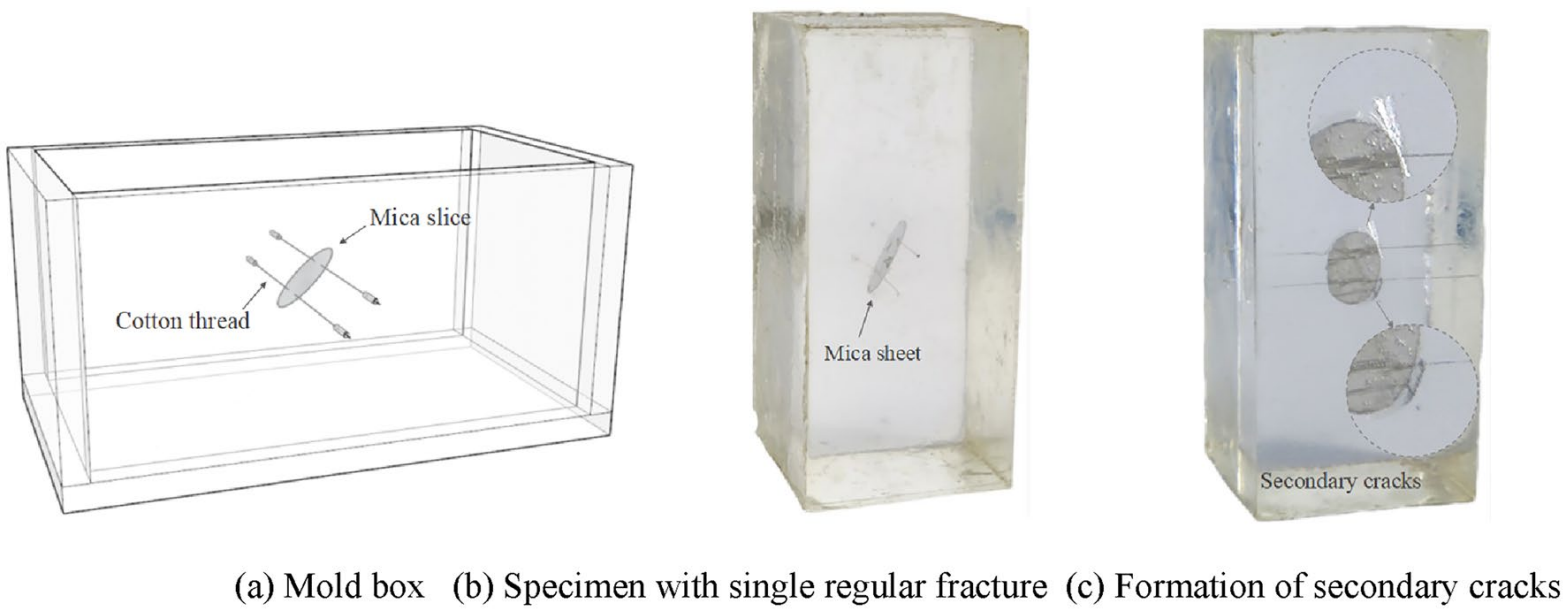
Preparation effect of transparent rock-like specimens with three-dimensional regular fractures.

Currently, the common method for preparing transparent rock-like specimens with three-dimensional fractures involves fixing mica sheets with the cotton thread inside a transparent mold box as shown in Fig. 7a, followed by pouring liquid resin to cure into a regular fractured specimen as shown in Fig. 7b. To determine the reasonable ratio for preparing regular fractured specimens, attempts were made to prepare transparent rock-like specimens with regular three-dimensional fractures at different angles using specimen ratios similar to the mechanical properties of hard rocks, specifically those exhibiting splitting and bursting failure modes. During the specimen preparation process, it was found that not any combination of ratios could successfully achieve the preparation of the regular fractured specimens. Under certain accelerator and hardener ratios, specifically those used for specimens with splitting and bursting failures, the resin curing process tends to be relatively intense. Due to the insertion of mica sheets, which act as foreign objects within the specimen, secondary cracks can form along the edges of the mica sheets during the heat-releasing curing process, as shown in Fig. 7c. Sometimes, these cracks may even extend through the entire specimen, preventing the successful preparation of the desired fracture structure.

**Figure 8:**
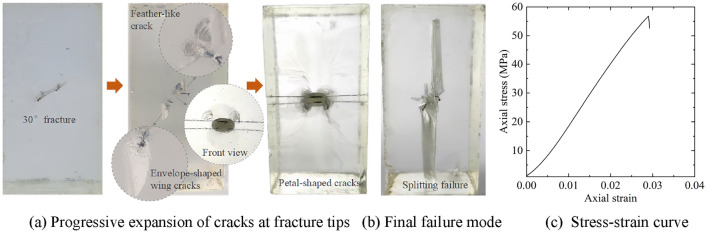
Fracture evolution process and stress–strain curve of the transparent rock-like specimen with a single three-dimensional regular fracture at 30° dip angle.

The preparation effects of transparent rock-like specimens with three-dimensional regular fractures under various ratio conditions are shown in Table 8. It is evident that under conditions of low and lower hardener ratios, medium and above accelerator ratios, or medium hardener ratios, medium and higher accelerator ratios, the specimens have good effects and a high success rate in preparation. Based on this ratio pattern, a successful preparation of transparent rock-like specimens with embedded mica sheets was achieved using a medium level of accelerator and hardener ratios. Figure 8a shows the progressive failure characteristics of the specimen with a single three-dimensional regular fracture at 30°dip angle under uniaxial compression conditions. Initially, feather-like cracks develop at the tips of the pre-existing fracture. With an increase in load, these feather-like cracks gradually extend into envelope-shaped wing cracks, then further expand to form petal-shaped cracks. Eventually, the specimen undergoes complete splitting failure, as illustrated in Fig. 8b. This represents the typical behavior of progressive expansion of cracks at three-dimensional fracture tip under uniaxial compression conditions. The stress–strain curve of the fractured specimen rapidly drops after peak stress, leading to a brittle failure, as shown in Fig. 8c. Therefore, using this composition to prepare transparent rock-like specimens with three-dimensional regular fractures is highly suitable for studying the fracturing evolution mechanism of the rock mass with three-dimensional regular fractures.

**Table 8:** Effects of specimen preparation for specimens with three-dimensional regular fractures under various mix proportion conditions.

A	B	Specimen preparation effect
Low and lower	Medium and above	The transparency of the specimen is high, no secondary cracks occur at the edges of the mica sheets, the success rate of prefabricated three-dimensional regular fractures is high, and the specimen preparation effect is good
Medium	Medium and higher	
Higher	Lower and above	There is a high probability of secondary cracks forming at the edges of the mica sheets, resulting in a low success rate of specimen preparation
Medium	High	
High	Higher and high	Secondary cracks occur at the edges of the mica sheets and expand intensely, sometimes even penetrating the entire specimen, making it very difficult to successfully prepare the specimen

## Random fractured specimens

**Figure 9:**
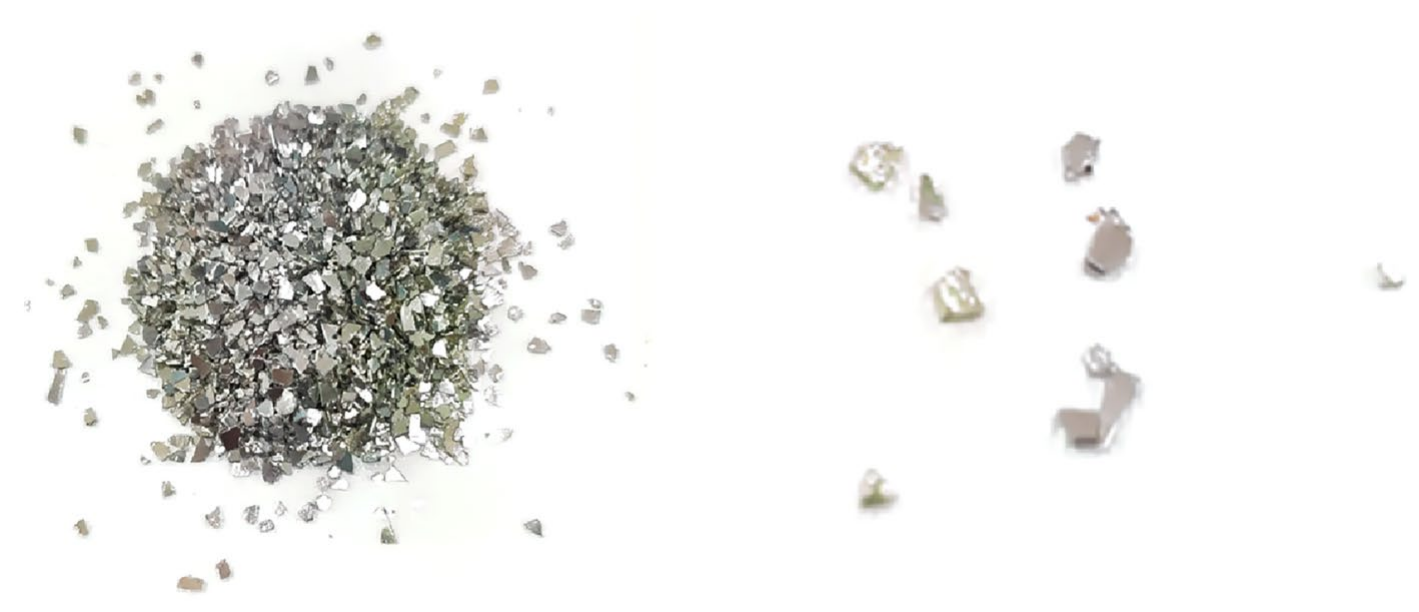
Irregular metal shavings.

In strict terms, the properties of three-dimensional fractures prepared using the cotton thread-fixed mica sheet method are considered as filled fractures, with mica sheets serving as the filling material. In contrast, the commonly developed three-dimensional fractures tend to be in a closed state. Additionally, the regular boundaries of the specimen box and the open boundaries determine that it is not possible to prepare arbitrary configuration fractures within the specimen. Lastly, when there are a significant number of pre-existing fractures inside the specimen, an excessive number of intersecting fixed cotton threads can affect the mechanical properties of the specimen. Based on the phenomenon of secondary cracks induced by mica sheets and the composition conditions, a method for preparing transparent rock-like specimens with self-induced random fractures inside is proposed. This method can achieve the preparation of three-dimensional random closed fractured specimens. Experimental results have shown that using irregular metallic shavings as foreign bodies for inducing fractures can ensure a higher fracture induction rate, as shown in Fig. 9.

Under different mixing ratios and the addition of accelerator and hardener, the results of preparing random fractured specimens vary significantly, as shown in Table 9. Under conditions where the reaction process is relatively slow (low and lower hardener ratio, medium and above accelerator ratio, or medium hardener ratio, medium and higher accelerator ratio), due to slow heat release, the waiting time for the test until metallic shavings are placed is long, and the success rate of inducing random fractures is very low. Under conditions where the reaction process is very intense (high hardener ratio, higher and high accelerator ratio), the specimen solidifies rapidly, and after the metallic shavings are added, they stop sinking at the top of the specimen, making it impossible to control the location of fractures. In severe cases, the intense curing reaction can cause the specimen to develop through fractures and split open, so the above-mentioned mixing conditions are not suitable. Preliminary experiments have shown that using the conditions with higher hardener ratio and lower to high accelerator ratio or medium hardener ratio and high accelerator ratio, which result in splitting or bursting failure, yields a higher success rate in inducing fractures through metallic shavings. Furthermore, the fracture does not significantly expand during the specimen curing process.

**Table 9:** Effects of specimen preparation for specimens with three-dimensional random fractures under various mix proportion conditions.

A	B	Specimen preparation effect
Low and lower	Medium and above	The heat release process during curing is slow, requiring a wait of more than 10 min, and the success rate of inducing fractures is very low
Medium	Medium and higher	
Higher	Lower and above	The curing time is 2 ~ 4 min, providing sufficient time to insert the metal shavings, and the success rate of inducing fractures is high
Medium	High	
High	Higher and high	The curing process is fast, making it difficult to successfully position the metal shavings. The heat release during curing is intense, and the specimen may even burst

**Figure 10:**
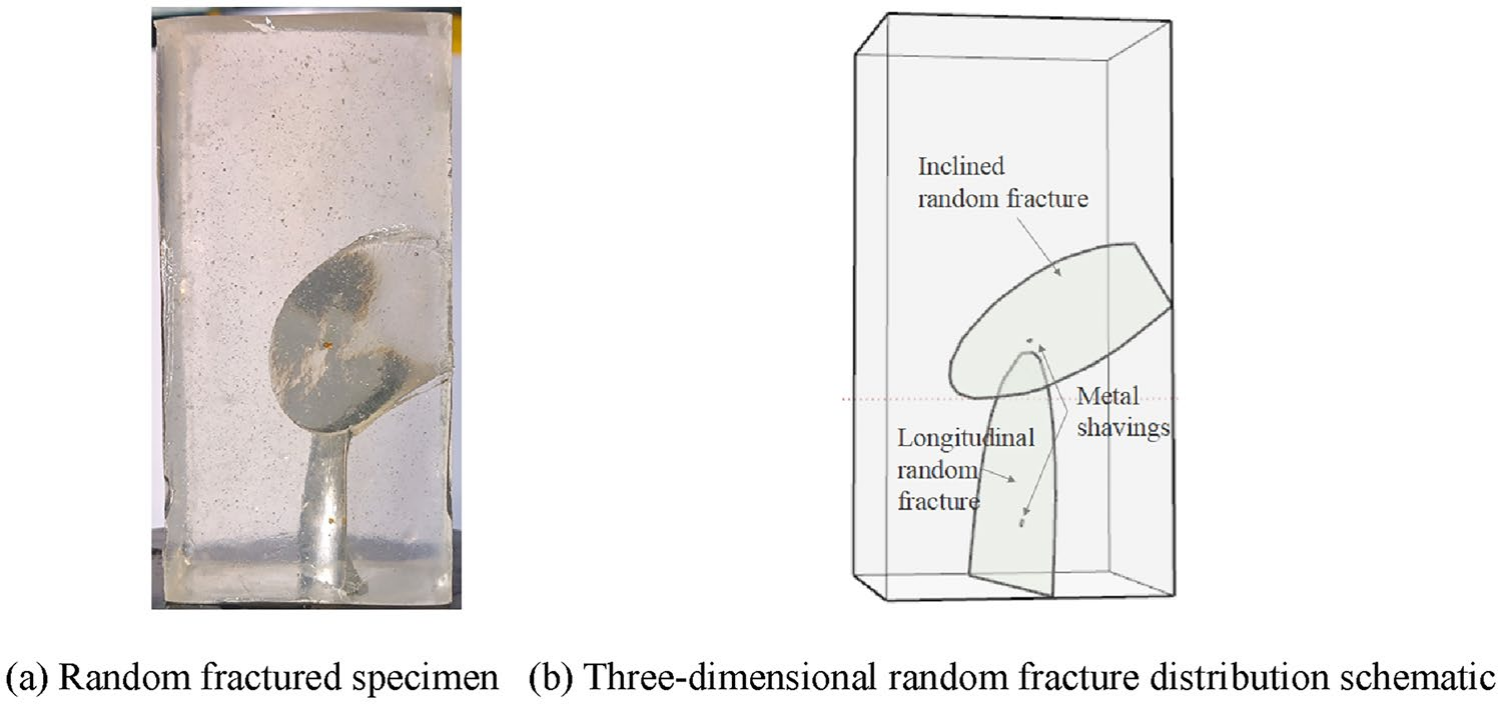
Transparent rock-like specimen with three-dimensional random fractures.

**Figure 11:**
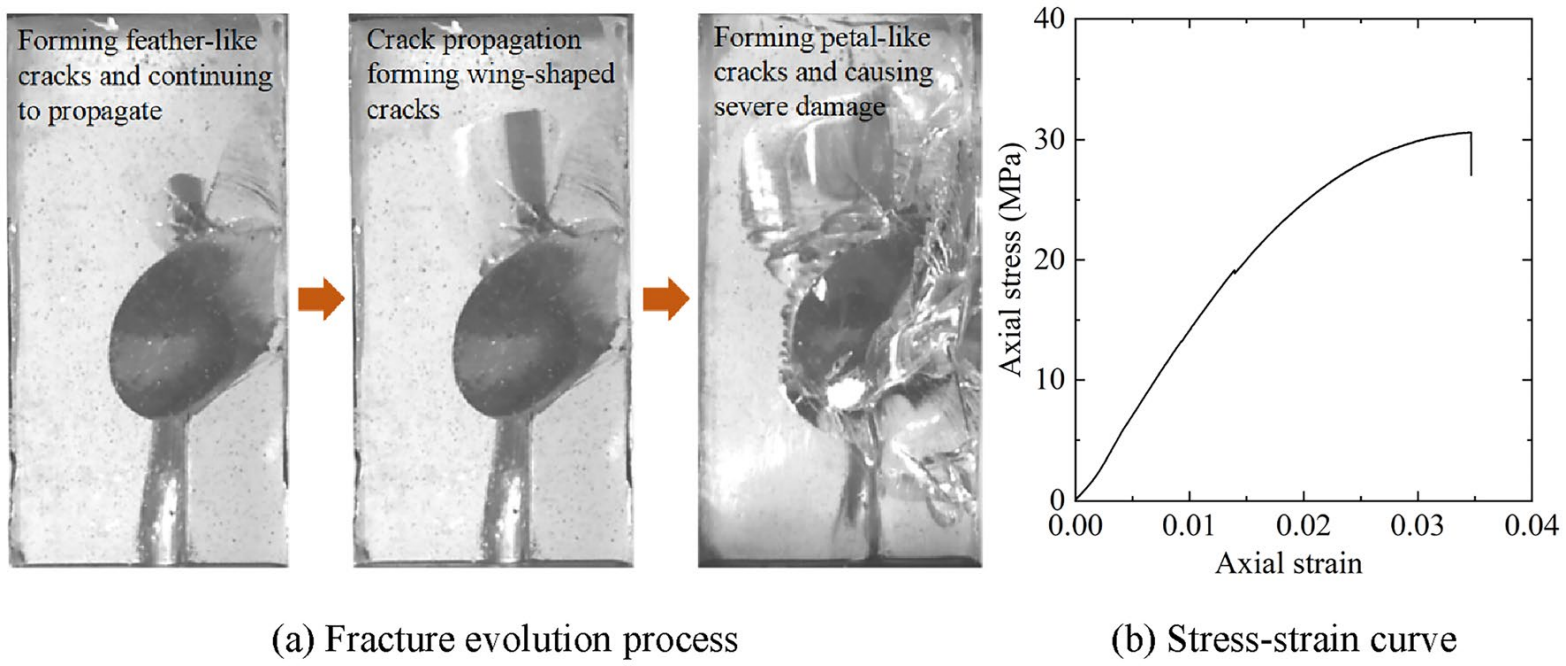
Fracture evolution process and stress–strain curve of the transparent rock-like specimen with three-dimensional random fractures.

In different proportioning conditions, the curing time varies. To ensure a high success rate of inducing random fractures, the state of the resin during the insertion of metal shavings needs to be controlled based on experimental phenomena. When adding metal shavings, the surface of the resin mixture should be viscous but not yet jelly-like. The shaving will move slowly and stop at a certain position in the specimen box as the curing reaction proceeds. By observing the movement speed of the shaving and adjusting the time of metal shavings insertion, the final resting position of the metal shaving can be controlled. This, in turn, induces random fractures during the heat release process of specimen curing, ultimately creating a transparent rock-like specimen with random fractures. Taking the random fractured specimen shown in Fig. 10 as an example, the specimen successfully induced two three-dimensional closed random fractures with two metal shavings. During the fracture generation process, due to the obstruction of the initially generated inclined random fracture, the longitudinal random fracture stopped expanding upon contact with it. This process of fracture generation can effectively simulate the formation pattern of fractures in actual rock masses. The progressive failure process and the stress–strain curve of this random fractured specimen under uniaxial compression are shown in Fig. 11a,b, respectively. Under uniaxial compression, the expansion pattern of the random fractures is similar to that of the three-dimensional regular fractures. The upper tip of the inclined random fracture expands to form feather-like cracks, which gradually evolve into wing-shaped and petal-shaped cracks as the load increases. Due to the influence of the longitudinal random fracture, the tip crack of the inclined random fracture does not extend downward. The longitudinal random fracture is nearly parallel to the loading direction and is also affected by the inclined random fracture, preventing the tip crack from extending upwards. When the load reaches its peak value, the tip crack of the inclined random fracture rapidly expands and connects with the longitudinal random fracture, causing the specimen to undergo splitting failure, displaying characteristics of brittle fracture. This process shows certain similarities to the progressive expansion characteristics of specimens with three-dimensional regular fractures. At the same time, it clearly reveals the interaction effect of the random fracture network and its control effect on the progressive failure of the specimen.

## Discussion

1. Due to the limitations of experimental conditions, this study only conducted low-temperature treatment at −25 ℃ for the specimens. However, previous studies have shown that low-temperature treatment below -30 ℃ can significantly increase the brittleness of transparent rock-like materials. Therefore, to further enhance the brittleness of transparent rock-like materials, explorations can be conducted on the basis of this study by further reducing the freezing temperature.
2. The current method of inducing the formation of random closed fractures using metal shavings can only control the location of fracture initiation, but the spatial orientation and size of the fractures are still not well controlled. Future research could focus on the size and shape of the inducing materials, in combination with fine adjustments to the accelerator ratio and hardener ratio content. This approach aims to explore further the main controlling factors of the orientation and size of random fractures. By doing so, it may be possible to achieve a more orderly generation of complex random fracture networks.
3. The uniaxial compressive strengths of the transparent rock-like materials formulated in this paper are high, which makes it difficult to apply them to large-scale model tests through the similarity theory, and is more suitable for the study of fractured rock damage mechanisms at the scale of indoor test specimens. In addition, the preparation of large-volume transparent rock-like specimens will face the bottleneck of large exothermic resin curing, serious internal damage of the specimen, and difficult to achieve the target structure specimen preparation, so the pursuit of the multi-angle high similarity between transparent rock-like materials and real rocks in terms of mechanical properties and failure modes, and their application in large-scale model tests need further in-depth research.

## Conclusions

In this paper, through an orthogonal experimental design and based on the uniaxial compression test and Brazilian split test results of transparent rock-like specimens, with the assistance of a high-speed camera system and SEM for experimental support, we systematically investigated the effects of resin, hardener, accelerator proportions, and freezing time on the mechanical properties of transparent rock-like specimens. The following conclusions were reached:

1. The influence of various factors on the uniaxial compressive strength and elastic modulus of transparent rock-like specimens, in descending order of impact, are the accelerator ratio, freezing time, and hardener ratio; whereas for tensile strength, the order is accelerator ratio, hardener ratio, and freezing time. As the accelerator ratio and freezing time increase, the compressive strength, tensile strength, and elastic modulus generally show a declining trend. With an increase in the hardener ratio, compressive strength and elastic modulus initially increase and then decrease. The effect of the hardener ratio on tensile strength is characterized by fluctuations, with a range of about 2 MPa, but overall, it still shows a trend of initially increasing and then decreasing.
2. Under uniaxial compressive load, transparent rock-like specimens exhibit four typical modes of failure: bursting failure, splitting failure, single inclined plane failure, and bulging failure. With the increase in the levels of hardener ratio and accelerator ratio, the failure mode gradually shifts from bulging to bursting. The accelerator ratio is the main factor controlling the failure mode of the specimens, with the increase of the accelerator ratio, the failure mode is gradually transformed from plastic to brittle failure; under the same level of accelerator ratio, the hardener ratio accelerates the process of transformation of the failure mode to brittle failure. The freezing time has a relatively minor effect on the overall trend of failure mode evolution. The compressive-tensile strength ratio of specimens undergoing splitting and bursting failures shows significant variability, with ranges of 6.22 to 13.9 and 9.27 to 19.21, respectively, exhibiting typical characteristics of brittle failure.
3. A method for creating self-induced random three-dimensional closed fractures within transparent rock-like specimens was proposed. This method provides technical support for the research on the interaction effects of random fracture networks and their control effects on the progressive failure of rock masses.
4. The proportioning principles for transparent rock-like specimens with different fracture structures were clarified. Specifically, it was found that under conditions of low and lower hardener ratios, medium and above accelerator ratios, or a combination of medium hardener and medium or higher accelerator ratios, the production of specimens with three-dimensional regular fractures is more effective and has a higher success rate. On the other hand, using higher hardener ratios, lower and above accelerator ratios, or a combination of medium hardener ratios and high accelerator ratios, increases the success rate of inducing random closed fractures through metal shavings.

## Data Availability

The data used to support the findings of this study are available from the corresponding author upon request.
